# ﻿A zoogeographical analysis of true bugs (Insecta, Heteroptera) from Uzbekistan

**DOI:** 10.3897/zookeys.1163.99414

**Published:** 2023-05-22

**Authors:** Lola Gandjaeva, Ikram Abdullaev, Abdulla Iskandarov, Komila Allabergenova, Saodat Yusupova, Gulkhayo Narimanova, Erkinboy Yusupboev, Sohiba Ibragimova, Sanjar Begliev, Khulkar Bobojonova

**Affiliations:** 1 Khorezm Mamun Academy, Markaz Str.,1, Khiva, Uzbekistan Khorezm Mamun Academy Khiva Uzbekistan; 2 Urgench State University, Khamid Alimjan Str.,14, Urgench, 220100, Uzbekistan Urgench State University Urgench Uzbekistan; 3 The Academic Lyceum of the Urgench branch of the Tashkent Medical Academy, Khamid Alimjan str.,14, Urgench, 220100, Uzbekistan The Academic Lyceum of the Urgench branch of the Tashkent Medical Academy Urgench Uzbekistan

**Keywords:** Abundance, distribution, faunistics, geography range, Karakalpakstan, Khorezm, Lower Amudarya, occurrence

## Abstract

The purpose of this study is to perform a zoogeographic analysis of terrestrial true bugs (Heteroptera) in the Lower Amudarya Territory, Uzbekistan. According to the findings of a literature review, there are 149 species of terrestrial hemipterans in the Territory. All hemipteran species, with the possible exception of five, have most likely been misidentified. Until now, reliable information on the zoogeography of true bug species in Uzbekistan’s north-western region has not been published. The composition of species, diversity, and the proportion of endemism vary greatly across the country’s zoogeographic regions. The Heteroptera fauna of the Khorezm and Karakalpakstan can be divided into four groups: most species belonging to the Palaearctic region, with 125 species constituting 83.89% of the fauna; the second group of Holarctic origin is characterized by no more than ten species, which constitutes 6.71%; the third consists of endemics, 13 species or 8.72%; and one species (0.67%) is cosmopolitan. Much more research is needed to investigate distributions in a more northern climate. The introduction of invasive Heteroptera to the north-western part of Uzbekistan will increase and deserves further consideration.

## ﻿Introduction

Heteroptera or true bugs are a large group with more than 40,000 species in approximately 50 families distributed across the world (Weirauch and Schuh 2011; Henry 2017a). In Russia, 760 species in 285 genera, and 35 families, are recorded (Vinokurov et al. 2010), however, more than 1250 species are distributed in Central Asia (Esenbekova 2013), and 700 species of true bugs are distributed in Uzbekistan (Animal World of Uzbekistan 2023).

The study of the fauna of true bugs by Central Asia region has been occurring for more than 170 years (Saprykin 2013). Many individuals have studied regional true bugs from 1995–2013 using the large, published Catalogue of Palaearctic Heteroptera (Catalogue of Palaearctic true bugs 2013).

The geographical distribution of Heteroptera from around the world has always been of interest to researchers (Latreille 1810; Leach 1815; Panizzi and Grazia 2015; Schuh and Weirauch 2020). Many research papers have been published recently, including Chandra and Kushwaha (2013); Samra et al. (2015); Vinokurov et al. (2015); Yasunaga (2016); Drapolyuk (2017); Henry (2017b); Oh et al. (2017); Kim and Jung (2018); Kuzhuget and Vinokurov (2018); Gapon (2019); Yazici (2020); Gandjaeva (2011, 2012a, b, 2020); Gandjaeva and Abdullaeva (2022a, b); Gandjaeva and Allabergenova (2022); Gandjaeva et al. (2019, 2020a, b, c, d, e, 2021, 2022a, b, c); Abdullaev et al. (2020a, b); Allabergenova and Gandzhaeva (2022); Yusupova and Gandjaeva (2022);Yusupova et al. (2022); Iskandarov et al. (2022).

Since the second half of the 19^th^ century, new descriptions of Central Asian species have been published regularly in the works of Yakovlev (1890); Oshanin (1891) and others. These researchers conducted route surveys in the Fergana Valley, Turkestan Ridge, Alay Range, and Alay Valley, as well as in Samarkand and Djizzakh. Approximately 384 species of true bugs were identified during expeditions, and their zoogeography was studied in Central Asia by prominent zoologists such as Oshanin (1891), who was the first scientist to investigate Heteropteran zoogeography and listed more than 530 species. In the 21^st^ century, many American scientists studied regional Heteroptera including Rider (2006, 2016); Hoebeke and Carter (2003); Bundy and McPherson (2018); Schuh and Weirauch (2020).

The literature on the fauna of terrestrial true bugs in different habitats of the Republic of Uzbekistan is meager. This lack of study also includes true bugs of Central Asia, mainly in the southern regions, which cover the territories of Samarkand, Bukhara, Tashkent, Andijan, Fergana, Kashkadarya, and Surkhandarya.

The purpose of the current paper is to explain database entries for the Lower Amudarya Heteroptera species, including brief geographic histories and original references. Every database should be a living document, with the ability to track changes regularly. Additional information on newly studied species is being added continuously (Gandjaeva 2011, 2012a, b, 2020; Gandjaeva et al. 2019, 2020a, b, c, d, e, 2021, 2022a, b, c; Abdullaev et al. 2020a, b; Allabergenova and Gandzhaeva 2022; Gandjaeva and Abdullaeva 2022a, b; Gandjaeva and Allabergenova 2022; Iskandarov et al. 2022; Yusupova and Gandjaeva 2022; Yusupova et al. 2022).

The goals of this study include classifying species ranges and conducting a zoogeographical analysis of the nation’s actual true bug fauna, as well as determining species compositions and distributions in various belts of the Khorezm region and Karakalpakstan Republic.

## ﻿Materials and methods

The study was conducted in a lowland area in the northwestern part of Uzbekistan along the lower sections of the Amudarya River: between 60' and 61' longitude and 41' and 42' latitude, at an altitude of 113–138 m above sea level. The vegetative cycle of plants lasts 200–210 days. The climate is continental, with an average annual precipitation of 80–90 mm, and average temperature ranges from -5 °C in January to 40 °C in July. The climate has been changing, and the temperature has risen in summer, reaching 50 °C in July (Gandjaeva 2019; Abdullaev et al. 2022; Ruzmetov et al. 2022). The usual alkali soils are meadow, meadow–marsh, and marsh–sandy. The area is located in the steppe zone, as well as in the southern portion of the Aral Sea and the western part of the Khorezm oasis. The historic Amudarya delta is made up of river sediments. Sand can be found on the sections connecting with Karakum in the west and southwest. Minerals include limestone, sand, clay, and other building materials (Khamraev 2003).

For the analysis, we used zoogeographical categories of the heteropteran species that had been recorded earlier. Approximately 180 specimens of Heteroptera indexed in the territory of the Lower Amudarya River and were identified to 149 species in 89 genera, and two infraorders. These species were deposited in the Zoological collections of the Zoology Institute (**ZIN**) of the Academy of Sciences of the Republic of Uzbekistan.

**Figure 1.**
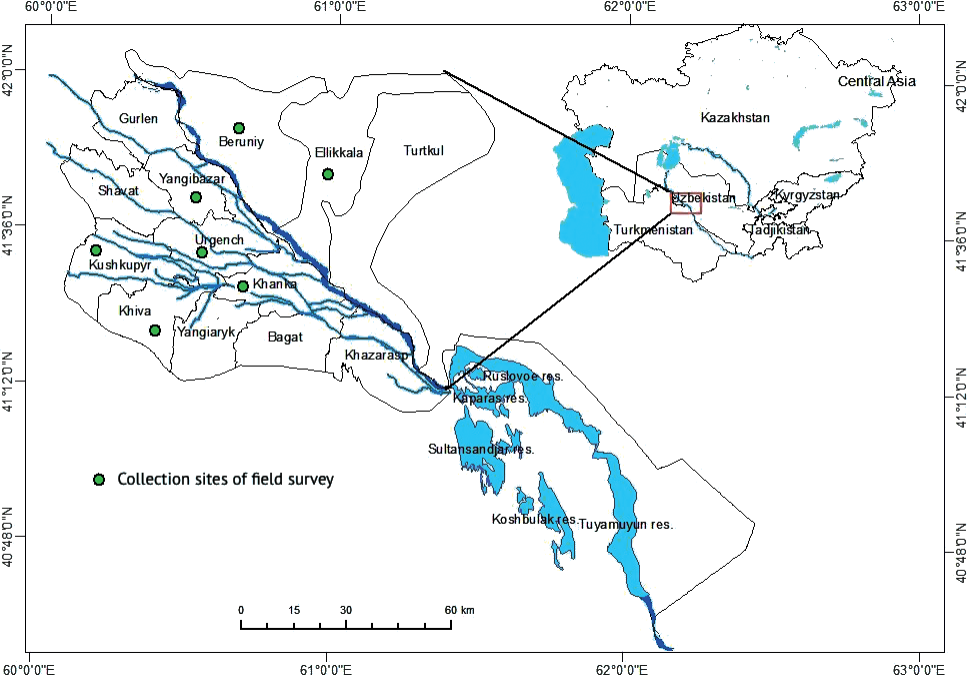
Geographical locations of the collection sites of terrestrial Heteroptera specimens in the Lower Amudarya Territory.

The study was carried out between 2007 and 2020 (see Gandjaeva 2011, 2012a, b, 2020; Gandjaeva et al. 2019, 2020a, b, c, d, e, 2021, 2022a, b, c; Abdullaev et al. 2020a, b; Allabergenova and Gandzhaeva 2022; Gandjaeva and Abdullaeva 2022a, b; Gandjaeva and Allabergenova 2022). Terrestrial Heteroptera were collected from various fields, including the agricultural farms “Odilbek,” “Amir Temur,” “Gulrukhbegim,” and “Oltin Kal’a” located in the Urgench district, “Dildora Bojimon” and “Buz Os Yep” agricultural farms, as well as the educational-experimental station of UrSU named “Uchkhoz” in Yangibazar district, “Ziroat-21” agricultural farm of Kushkupir district, “Raximbergan Xoji Anbar” in Khiva district, “Otabek garchak” and “Gulkand Istikbolli bog’i” in Khanka district and natural landscapes in the Khorezm region, as well as “Zaripboy,” “Kilchinok,” and “Yangiyer” agricultural farms in Ellikkala district of the Republic of Karakalpakstan and “Badai Tugai Nature Reserve,” Karatau mountain in the Beruniy district of the Republic of Karakalpakstan (Gandjaeva et al. 2021). The geographical locations of the sites are shown in Fig. 1.

The zoogeographic analysis of identified species in the Lower Amudarya was based on zoogeographical nomenclature by Emelyanov (1974). In brief, geographic longitude was used to establish the zone along its meridional boundaries.

The descriptive area nomenclature utilized in this work uses the concepts of physical geography and applies two axis coordinates: the latitudinal axis runs from north to south and is critical because it is used to determine climatic conditions of the distributed species, especially temperature; the longitudinal axis runs from west to east. In some species, the range coincides with the boundaries of the landscape zone and is labeled as Arctic (polar deserts, tundra), boreal (taiga), subboreal (broad-leaved forests), subtropical and tropical (evergreen forests) (Lopatin and Meleshko 2016).

We used the basic data on the geographic distribution of these species from the Catalogue “Heteroptera of the Palaearctic” Volumes I–VI, published by the Netherlands Entomological Society, Amsterdam (NES) (1995–2013) (Catalogue of Palaearctic true bugs 2013) to describe the analysis of the zoogeographic areas of terrestrial Heteroptera (Aukema et al. 2013) the database is continually updated.

An analysis of the occurrence and abundance of species on cultivated and wild plants were carried out by observing 50–100 plant specimens every day along the diagonal of the fields. The number of adult bugs, larvae of all ages, and egg clutches was recorded (Gandjaeva et al. 2021).

The number of species and their occurrence was calculated using the formula devised by Dajoz (2000):

F(%) = 100 × (Pi/P)

where Pi refers to the species that was found; P is an absolute number.

Species are divided into four groups based on their frequency of occurrence:

Constantly occurring species: F ≥ 50%

Often occurring species: 25% < F < 50%

Additional occurring species: 5% ≤ F < 25%

Rarely occurring species: F < 5%

The dynamics of the abundance of species was calculated using the formula of Zaime and Gautier (1989):

Ar(%) = 100 × (Ni/N)

where Ni is the coefficient of special observable species; N is the absolute number of all observable species.

The analyses of the dynamics of the numbers of species are also divided into four groups:

Abundant: Ar ≥ 10

Frequent: 5 ≤ Ar < 10

Some: 1 ≤ Ar < 5

Few: Ar < 1

## ﻿Results and discussion

Checklists of Heteroptera for the Khorezm region and Karakalpakstan Republic were published more than 20 years ago. Khamraev (2003) and Kulumbetova (1998a, b, c, 1999) listed several species found to the north of Uzbekistan and, respectively, but some species have yet to be discovered while others are rare or migratory.

**Figure 2.**
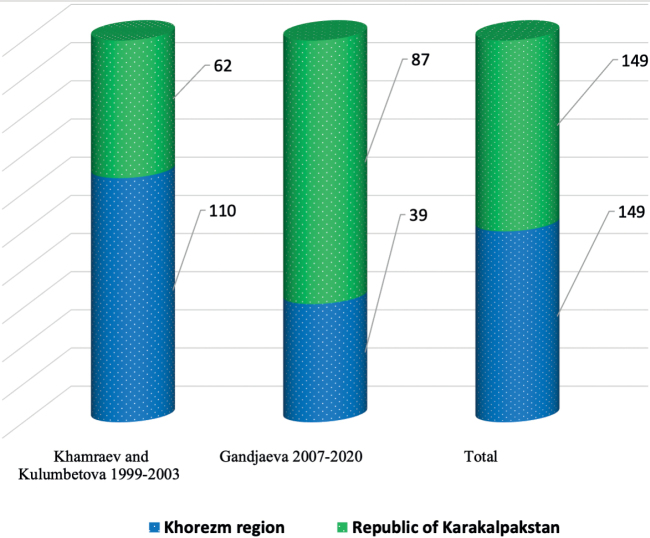
Numbers of terrestrial Heteroptera recorded in the regions of the northern part of Uzbekistan.

We carried out a comparative analysis of the lists of regional faunas using the data from Khamraev (2003) in the Khorezm Region and Kulumbetova (1998a, b, c, 1999) in the Republic of Karakalpakstan, which allowed us to determine regional features of the fauna in the Lower Amudarya (Table 1). Based on taxonomic distribution, this method enables the collection of data about species complexes with various zoogeographical characteristics (Table 1; Fig. 2). To classify the areas of the Lower Amudarya, information from Gandjaeva et al. (2021) was used (Fig. 2).

**Table 1.** Checklist of the terrestrial Heteroptera from the Lower Amudarya (2007–2020).

Taxon		Family	Occurrence	Abundance	Distribution	Zoogeographic categories	References
1	*Anthocorispilosus* (Jakovlev, 1877)	Anthocoridae Fieber, 1837	+	F	***	SA	Khamraev (2003); Gandjaeva et al. (2021)
2	*Oriusniger* (Wolff, 1811)		++	FR	0	TP	Khamraev (2003); Kulumbetova (1999); Gandjaeva et al. (2021)
3	*Oriusribauti* (Wagner, 1952)		+	F	***	P	Khamraev (2003); Gandjaeva et al. (2021)
4	*Oriusalbidipennis* (Reuter, 1884)		+	S	**	TP	Kulumbetova (1999); Gandjaeva et al. (2021)
5	*Nabisferus* (Linnaeus, 1758)	Nabidae Costa, 1852	++	FR	0	TP	Khamraev (2003); Kulumbetova (1999); Gandjaeva et al. (2021)
6	*Nabispalifer* (Seidenstücker, 1954)		+	F	***	TS	Khamraev (2003); Gandjaeva et al. (2021)
7	*Nabisviridis* (Brullé, 1839)		+	F	***	SA	Khamraev (2003); Gandjaeva et al. (2021)
8	*Nabisrugosus* (Linnaeus, 1758)		++	FR	***	SA	Khamraev (2003); Gandjaeva et al. (2021)
9	*Nabisremanei* (Kerzhner, 1962)		+	F	**	ChCA	Kulumbetova (1999); Gandjaeva et al. (2021)
10	*Nabissareptanus* (Dohrn, 1862)		+	F	***	TP	Khamraev (2003); Gandjaeva et al. (2021)
11	*Prostemmasanguineum* (Rossi, 1790)		+	F	**	PA	Kulumbetova (1999); Gandjaeva et al. (2021)
12	*Deraeocorispunctulatus* (Fallén, 1807)	Miridae Hahn, 1833	++	FR	0	P	Khamraev (2003); Kulumbetova (1999); Gandjaeva et al. (2021)
13	*Deraeocorisserenus* (Douglas & Scott, 1868)		++	FR	** **	W	Gandjaeva et al. (2021)
14	*Adelphocorislineolatus* (Coeze, 1778)		+++	A	0	TP	Khamraev (2003); Kulumbetova (1999); Gandjaeva et al. (2021)
15	*Adelphocorisseticornis* (Fabricius, 1775)		+++	A	** **	W	Gandjaeva et al. (2021)
16	*Agnocorisrubicundus* (Fallen, 1807)		++	FR	***	TP	Khamraev (2003); Gandjaeva et al. (2021)
17	*Brachycoleusdecolor* (Reuter, 1887)		++	FR	***	W	Khamraev (2003); Gandjaeva et al. (2021)
18	*Lyguspratensis* (Linnaeus, 1758)		+++	A	0	SA	Khamraev (2003); Kulumbetova (1999); Gandjaeva et al. (2021)
19	*Lygusgemellatus* (Herrich-Schäffer, 1835)		+++	A	0	TP	Khamraev (2003); Kulumbetova (1999); Gandjaeva et al. (2021)
20	*Lyguspachycnemis* (Reuter, 1879)		+++	A	***	TNT	Khamraev (2003); Gandjaeva et al. (2021)
21	*Lygusrugulipennis* (Poppius, 1911)		+++	A	** **	TP	Gandjaeva et al. (2021)
22	*Lyguspunctatus* (Zetterstedt, 1838)		+++	A	** **	TP	Gandjaeva et al. (2021)
23	*Megacoelumbrevirostre* (Reuter, 1879)		++	FR	***	TS	Khamraev (2003); Gandjaeva et al. (2021)
24	*Orthopsbasalis* (Costa, 1853)		++	FR	***	SA	Khamraev (2003); Gandjaeva et al. (2021)
25	*Orthopskalmi* (Linnaeus, 1758)		++	FR	0	SA	Khamraev (2003); Kulumbetova (1999); Gandjaeva et al. (2021)
26	*Polymerusvulneratus* (Panzer, 1806)		+++	A	0	TP	Khamraev (2003); Kulumbetova (1999); Gandjaeva et al. (2021)
27	*Polymeruscognatus* (Fieber, 1858)		+++	A	**	TP	Kulumbetova (1999); Gandjaeva et al. (2021)
28	*Notostiraelongata* (Geoffroy, 1785)		++	FR	** **	SA	Gandjaeva et al. (2021)
29	*Megaloceroearecticornis* (Geoffroy, 1785)		++	FR	** **	W	Gandjaeva et al. (2021)
30	*Stenodemacalcaratum* (Fallen, 1807)		+++	A	0	TP	Khamraev (2003); Gandjaeva et al. (2021)
31	*Stenodematripsinosa* (Reuter, 1904)		+++	A	***	TP	Khamraev (2003); Gandjaeva et al. (2021)
32	*Stenodemalaevigata* (Linnaeus, 1758)		+++	A	***	PA	Khamraev (2003); Gandjaeva et al. (2021)
33	*Stenodematuranica* (Reuter, 1904)		++	FR	0	NC	Khamraev (2003); Kulumbetova (1999); Gandjaeva et al. (2021)
34	*Trigonotylusruficornis* (Geoffroy, 1785)		++	FR	0	PA	Khamraev (2003); Kulumbetova (1999); Gandjaeva et al. (2021)
35	*Trigonotyluspulchellus* (Hahn, 1834)		++	FR	** **	P	Gandjaeva et al. (2021)
36	*Orthotyluseleagni* (Jakovlev, 1881)		++	FR	***	TS	Khamraev (2003); Gandjaeva et al. (2021)
37	*Orthotylusflavosparsus* (Sahlberg, 1841)	Miridae Hahn, 1833	++	FR	***	TP	Khamraev (2003); Gandjaeva et al. (2021)
38	*Campylommaannulicorne* (Signoret, 1865)		++	FR	**	P	Kulumbetova (1999); Gandjaeva et al. (2021)
39	*Campylommadiversicornis* (Reuter, 1878)		+++	A	***	NS	Khamraev (2003); Gandjaeva et al. (2021)
40	*Campylommaverbasci* (Meyer-Dur, 1843)		+++	A	**	PA	Kulumbetova (1999); Gandjaeva et al. (2021)
41	*Camptotylideaalba* (Reuter, 1879)		++	FR	***	TNT	Khamraev (2003); Gandjaeva et al. (2021)
42	*Camptotylusmeyeri* (Frey-Gessner, 1863)		++	FR	***	NS	Khamraev (2003); Gandjaeva et al. (2021)
43	*Europiellaalpina* (Reuter, 1875)		++	FR	** **	TP	Gandjaeva et al. (2021)
44	*Heterocapillustigripes* (Meyer & Dur, 1852)		+	F	*	SA	Gandjaeva et al. (2021)
45	*Macrotylusherrichi* (Reuter, 1873)		+	F	*	SA	Gandjaeva et al. (2021)
46	*Tuponiaelegans* (Jakovlev, 1867)		++	FR	***	SA	Khamraev (2003); Gandjaeva et al. (2021)
47	*Tuponiapallida* (Jakovlev, 1867)		++	FR	***		Khamraev (2003); Gandjaeva et al. (2021)
48	*Tuponiaroseipennis* (Reuter, 1889)		++	FR	***	ChCA	Khamraev (2003); Gandjaeva et al. (2021)
49	*Tarajalabrevicornis* (Reuter, 1879)		–	–	–	–	Khamraev (2003)
50	*Monosteiradiscoidalis* (Jakovlev, 1883)	Tingidae Laporte, 1832	+	F	0	SA	Khamraev (2003); Kulumbetova (1999); Gandjaeva et al. (2021)
51	*Stephanitispyri* (Fabricius, 1775)		+	F	** **	P	Gandjaeva et al. (2021)
52	*Tingisleptochila* (Horvath, 1906)		+	F	***	ITCA	Khamraev (2003); Gandjaeva et al. (2021)
53	*Stenolemusbogdanovi* (Oshanin, 1896)	Reduviidae Latreille, 1807	+	F	***	TS	Khamraev (2003); Gandjaeva et al. (2021)
54	*Coranusaegyptius* (Fabricius, 1775)		++	FR	***	SA	Khamraev (2003); Gandjaeva et al. (2021)
55	*Coranussubapterus* (De Geer, 1773)		++	FR	**	NS	Kulumbetova (1999); Gandjaeva et al. (2021)
56	*Rhynocorismonticola* (Oshanin, 1870)		++	FR	***	TS	Khamraev (2003); Gandjaeva et al. (2021)
57	*Rhinocorisnigronitens* Reuter, 1881		++	FR	***	TS	Khamraev (2003); Gandjaeva et al. (2021)
58	*Vachiriadeserta* (Becker,1867)		+	F	** **	ITCA	Gandjaeva et al. (2021)
59	*Ectomocorisululans* (Rossi, 1807)		+	F	***	ETPE	Khamraev (2003); Gandjaeva et al. (2021)
60	*Reduviustestaceus* (Herrich-Schäffer, 1845)		+	S	***	TS	Gandjaeva et al. (2021)
61	*Reduviusdisciger* (Horváth, 1896)		+	F	***	TS	Khamraev (2003); Gandjaeva et al. (2021)
62	*Reduviuschristophi* (Jakovlev, 1874)		+	S	0	TS	Khamraev (2003); Kulumbetova (1999); Gandjaeva et al. (2021)
63	*Reduviusfedtschenkianus* (Oshanin, 1871)		+	F	0	TNT	Khamraev (2003); Kulumbetova (1999); Gandjaeva et al. (2021)
64	*Reduviussemenovi* (Jakovlev, 1885)	Reduviidae Latreille, 1807	+	F	***	TNT	Khamraev (2003); Gandjaeva et al. (2021)
65	*Reduviuselegans* (Jakovlev, 1885)		++	FR	***	TNT	Khamraev (2003); Gandjaeva et al. (2021)
66	*Oncocephalusbrachymerus* (Reuter, 1882)		++	FR	***	TS	Khamraev (2003); Gandjaeva et al. (2021)
67	*Oncocephalustermezanus* (Kiritshenko, 1914)		++	FR	**	ITCA	Kulumbetova (1999); Gandjaeva et al. (2021)
68	*Camptopuslateralis* (German, 1817)	Alydidae Amyot & Serville, 1843	+	F	0	SA	Khamraev (2003); Kulumbetova (1999); Gandjaeva et al. (2021)
69	*Megalotomusornaticeps* (Stål, 1858)		+	F	**	NS	Kulumbetova (1999); Gandjaeva et al. (2021)
70	*Centrocorisvolxemi* (Puton, 1878)	Coreidae Leach, 1815	+	F	***	TS	Khamraev (2003); Gandjaeva et al. (2021)
71	*Coreusmarginatus* (Linnaeus, 1758)		+	S	***	TP	Khamraev (2003); Gandjaeva et al. (2021)
72	*Enoplopseversmanni* (Jakovlev, 1881)		+	F	***	T	Khamraev (2003); Gandjaeva et al. (2021)
73	*Bathysolennubilus* (Fallen, 1807)		+	F	**	TS	Kulumbetova (1999); Gandjaeva et al. (2021)
74	*Bothrostethusannulipes* (Herrich-Schäffer, 1835)		+	S	**	TS	Kulumbetova (1999); Gandjaeva et al. (2021)
75	*Coriomerisvitticollis* (Reuter, 1900)		+	F	0	TS	Khamraev (2003); Kulumbetova (1999); Gandjaeva et al. (2021)
76	*Brachycarenustigrinus* (Schilling, 1829)	Rhopalidae Amyot & Serville, 1843	++	FR	0	TP	Khamraev (2003); Kulumbetova (1999); Gandjaeva et al. (2021)
77	*Chorosomaschillingi* (Schilling, 1829)		++	FR	***	SA	Khamraev (2003); Gandjaeva et al. (2021)
78	*Corizuslimbatus* (Rey, 1887)		+++	A	0	SA	Khamraev (2003); Kulumbetova (1999); Gandjaeva et al. (2021)
79	*Corizustetraspilus* (Horvath, 1917)		+++	A	**	NS	Khamraev (2003); Kulumbetova (1999); Gandjaeva et al. (2021)
80	*Corizushyoscyami* (Linnaeus, 1758)		+++	A	***	TP	Khamraev (2003); Gandjaeva et al. (2021)
81	*Maccevethuspersicus* (Jakovlev, 1882)		++	FR	***	TS	Khamraev (2003); Gandjaeva et al. (2021)
82	*Liorhyssushyalinus* (Fabricius, 1794)		++	FR	0	C	Khamraev (2003); Kulumbetova (1999); Gandjaeva et al. (2021)
83	*Rhopalusparumpunctatus* (Schilling, 1829)		++	FR	***	TP	Khamraev (2003); Gandjaeva et al. (2021)
84	*Rhopalusdistinctus* (Signoret, 1859)		++	FR	***	TS	Khamraev (2003); Gandjaeva et al. (2021)
85	*Stictopleurusunicolor* (Jakovlev, 1873)		++	FR	***	W	Khamraev (2003); Gandjaeva et al. (2021)
86	*Dicranocephalusmarginatus* (Ferrari, 1874)	Stenocephalidae Dallas, 1852	+	F	0	TS	Khamraev (2003); Kulumbetova (1999); Gandjaeva et al. (2021)
87	*Dicranocephalusferghanensis* (Horváth, 1887)		+	F	0	TS	Khamraev (2003); Kulumbetova (1999); Gandjaeva et al. (2021)
88	*Artheneisalutacea* (Fieber, 1861)	Artheneidae Stål, 1872	+	S	***	W	Khamraev (2003); Gandjaeva et al. (2021)
89	*Geocorisater* (Fabricius, 1787)	Geocoridae Baerensprung, 1860	++	FR	**	TP	Kulumbetova (1999); Gandjaeva et al. (2021)
90	*Geocorisarenarius* (Jakovlev, 1867)		+	F	**	NS	Kulumbetova (1999); Gandjaeva et al. (2021)
91	*Geocorisdispar* (Waga, 1839)		++	FR	**	W	Kulumbetova (1999); Gandjaeva et al. (2021)
92	*Geocorislapponicus* (Zetterstedt, 1838)		+	F	** **	P	Gandjaeva et al. (2021)
93	*Geocorisfedtschenkoi* (Reuter, 1885)		+	F	***	NS	Khamraev (2003); Gandjaeva et al. (2021)
94	*Geocorisscutellatus* (Montandon, 1907)		+	F	***	KNTIT	Khamraev (2003); Gandjaeva et al. (2021)
95	*Engistussalinus* (Jakovlev, 1874)		+	F	***	TS	Khamraev (2003); Gandjaeva et al. (2021)
96	*Engistusexsanguis* (Stál, 1872)		++	FR	***	TS	Khamraev (2003); Gandjaeva et al. (2021)
97	*Henestarishalophilus* (Burmeister, 1835)		+	F	***	W	Khamraev (2003); Gandjaeva et al. (2021)
98	*Lygaeusequestris* (Linnaeus, 1758)	Lygaeidae Schilling, 1829	++	FR	0	TP	Khamraev (2003); Kulumbetova (1999); Gandjaeva et al. (2021)
99	*Spilostethusrubriceps* (Horvath, 1899)		+	F	0	TS	Khamraev (2003); Kulumbetova (1999); Gandjaeva et al. (2021)
100	*Spilostethuspandurus* (Scopoli, 1763)		+	F	**	TS	Kulumbetova (1999); Gandjaeva et al. (2021)
101	*Nysiusgraminicola* (Kolenati, F.A., 1845)		++	FR	***	SA	Khamraev (2003); Gandjaeva et al. (2021)
102	*Oxycarenuspallens* (Herrich-Schäffer, 1850)		+	S	***	SA	Khamraev (2003); Gandjaeva et al. (2021)
103	*Ortholomuspunctipennis* (Herrich-Schäffer, 1850)		++	FR	***	P	Khamraev (2003); Gandjaeva et al. (2021)
104	*Beosusquadripunctatus* (Muller, 1766)	Rhyparochromidae Amyot & Serville, 1843	++	FR	**	SA	Kulumbetova (1999); Gandjaeva et al. (2021)
105	*Bleteogonusbeckeri* (Frey-Gessner, 1863)		+	F	**	TS	Kulumbetova (1999); Gandjaeva et al. (2021)
106	*Emblethisgriseus* (Wolff, 1802)		+	F	0	SA	Khamraev (2003); Kulumbetova (1999); Gandjaeva et al. (2021)
107	*Emblethisverbasci* (Fabricius, 1803)		+	F	0	SA	Khamraev (2003); Kulumbetova (1999); Gandjaeva et al. (2021)
108	*Emblethisciliatus* (Horváth, 1875)		+	F	0	SA	Khamraev (2003); Kulumbetova (1999); Gandjaeva et al. (2021)
109	*Emblethisdenticollis* (Horváth, 1878)		+	F	***	P	Khamraev (2003); Gandjaeva et al. (2021)
110	*Emblethisdilaticollis* (Jakovlev, 1874)		–	–	–	–	Kulumbetova (1999)
111	*Hyalocorispilicornis* (Jakovlev, 1874)		+	S	0	TS	Khamraev (2003); Kulumbetova (1999); Gandjaeva et al. (2021)
112	*Lamprodemamaura* (Fabricius, 1803)		++	FR	0	W	Khamraev (2003); Kulumbetova (1999); Gandjaeva et al. (2021)
113	*Aethuspilosulus* (Klug, 1845)	Cydnidae Billberg, 1820	+	F	0	TS	Khamraev (2003); Kulumbetova (1999); Gandjaeva et al. (2021)
114	*Aethusnigronervosus* (Melichar, 1906)		–	–	–	–	Khamraev (2003)
115	*Byrsinusfossor* (Mulsant & Rey, 1866)		+	F	0	TP	Khamraev (2003); Kulumbetova (1999); Gandjaeva et al. (2021)
116	*Microporusvirgata* (Fabricius, 1794)		–	–	–	–	Khamraev (2003)
117	*Microporusnigrita* (Fabricius, 1794)		+	F	**	ETPE	Gandjaeva et al. (2021)
118	*Stibaropushohlbecki* (Kiritshenko, 1912)		+	F	**	TNT	Kulumbetova (1999); Gandjaeva et al. (2021)
119	*Sehirusmorio* (Linnaeus, 1761)		+	F	***	W	Khamraev (2003); Gandjaeva et al. (2021)
120	*Amaurocoriscandidus* (Horváth, 1889)		+	F	***	TS	Khamraev (2003); Gandjaeva et al. (2021)
121	*Aeliaacuminata* (Linnaeus, 1758)	Pentatomidae Leach, 1815	+++	A	**	W	Kulumbetova (1999); Gandjaeva et al. (2021)
122	*Aeliafurcula* (Fieber, 1868)		+++	A	***	TS	Khamraev (2003); Gandjaeva et al. (2021)
123	*Aeliamelanota* (Fieber, 1868)		+++	A	**	TS	Kulumbetova (1999); Gandjaeva et al. (2021)
124	*Brachynemagermari* (Kalenati, 1846)		++	FR	0	TP	Khamraev (2003); Kulumbetova (1999); Gandjaeva et al. (2021)
125	*Carpocorispudicus* (Poda, 1761)		++	FR	***	P	Khamraev (2003); Gandjaeva et al. (2021)
126	*Carpocorisfuscispinus* (Boheman, 1851)		++	FR	0	W	Khamraev (2003); Kulumbetova (1999); Gandjaeva et al. (2021)
127	*Palomenaprasina* (Linnaeus, 1761)		+++	A	** **	SA	Gandjaeva et al. (2021)
128	*Dolycorispenicillatus* (Horváth, 1904)		+++	A	0	TS	Khamraev (2003); Kulumbetova (1999); Gandjaeva et al. (2021)
129	*Desertomenidaquadrimaculata* (Horváth, 1892)		+++	A	***	NS	Khamraev (2003); Gandjaeva et al. (2021)
130	*Desertomenidaalbula* (Kiritshenko, 1914)		+++	A	***	TS	Khamraev (2003); Gandjaeva et al. (2021)
131	*Derulalongipennis* (Oshanin, 1871)		+	F	** **	TP	Gandjaeva et al. (2021)
132	*Apodiphusintegriceps* (Horváth, 1888)		+++	A	0	TS	Khamraev (2003); Kulumbetova (1999); Gandjaeva et al. (2021)
133	*Cellobiusabdominalis* (Jakovlev, 1885)		++	FR	***	NS	Khamraev (2003); Gandjaeva et al. (2021)
134	*Codophilavaria* (Fabricius, 1787)		++	FR	***	SA	Khamraev (2003); Gandjaeva et al. (2021)
135	*Holcostethusnitidus* (Kiritshenko, 1914)		++	FR	***	TNT	Khamraev (2003); Gandjaeva et al. (2021)
136	*Holcostethusstrictusvernalis* (Wolff, 1804)	Pentatomidae Leach, 1815	++	FR	**	P	Kulumbetova (1999); Gandjaeva et al. (2021)
137	*Menaccarusdeserticola* (Jakovlev, 1900)		++	FR	***	TS	Khamraev (2003); Gandjaeva et al. (2021)
138	*Eurydemaornata* (Linnaeus, 1758)		+++	A	***	SA	Khamraev (2003); Gandjaeva et al. (2021)
139	*Eurydemaoleracae* (Linnaeus, 1758)		+++	A	** **	SA	Gandjaeva et al. (2021)
140	*Eurydemawilkinsi* (Distant, 1879)		+++	A	*	NS	Gandjaeva et al. (2021)
141	*Eurydemaventralis* (Kolenati, 1846)		+++	A	** **	SA	Gandjaeva et al. (2021)
142	*Eurydemamaracandica* (Oshanin, 1871)		+++	A	**	NS	Kulumbetova (1999); Gandjaeva et al. (2021)
143	*Graphosomalineatum* (Linnaeus, 1758)		++	FR	***	SA	Khamraev (2003); Gandjaeva et al. (2021)
144	*Graphosomaconsimile* (Horvath, 1903)		++	FR	***	TS	Khamraev (2003); Gandjaeva et al. (2021)
145	*Tarisaelevata* (Reuter, 1901)		++	FR	***	TS	Khamraev (2003); Gandjaeva et al. (2021)
146	*Tarisasubspinosa* (*Germar*, *1839*)		++	FR	***	TP	Khamraev (2003); Gandjaeva et al. (2021)
147	*Tarisavirescens* (Herrich-Schäffer, 1851)		++	FR	***	NS	Khamraev (2003); Gandjaeva et al. (2021)
148	*Tarisapallescens* (Jakovlev, 1871)		++	FR	***	TS	Khamraev (2003); Gandjaeva et al. (2021)
149	*Sciocorishelferi* (Fieber, 1851)		–	–	–	–	Kulumbetova (1999)
150	*Eurygasterintegriceps* (Puton, 1881)	Scutelleridae Leach, 1815	++	FR	0	P	Khamraev (2003); Kulumbetova (1999); Gandjaeva et al. (2021)
151	*Odontotarsusimpictus* (Jakovlev, 1886)		+	F	0	TS	Khamraev (2003); Kulumbetova (1999); Gandjaeva et al. (2021)
152	*Odontotarsusangustatus* (Jakovlev 1883)		+	F	***	TS	Khamraev (2003); Gandjaeva et al. (2021)
153	*Scantiusaegyptius* (Linnaeus, 1758)	Pyrrhocoridae Amyot & Serville, 1843	+	F	0	NS	Khamraev (2003); Kulumbetova (1999); Gandjaeva et al. (2021)
154	*Pyrrhocorisapterus* (Linnaeus, 1758)		++	FR	0	W	Khamraev (2003); Kulumbetova (1999); Gandjaeva et al. (2021)
**Total number of species: 154**							

**Symbols and abbreviations used in the table Occurrence**: constantly occurring species **(CO)**: ++++; **o**ften occurring species **(OO)**: +++; additional occurring species **(AO)**: ++; Rarely occurring species **(RO)**: +. **Abundance**: Abundant: **A**; frequent: **FR**; some: **S**; few: **F**. **Distribution**: 0 – species presence; – species presence not confirmed * – previously unregistered species for Uzbekistan; ** – previously unregistered species for the Khorezm region; *** – previously unregistered species for the Republic of Karakalpakstan; ** ** – previously unregistered species for the Khorezm region and the Republic of Karakalpakstan. **Zoogeographical categories C** – Cosmopolitan; **TP** – Trans-Palaearctic; **P** – Pancontinental; **ETPE** – Ethiopia – Trans-Palaearctic – Eastern; **SA** – Super-Atlantic; **W** – The Western; **PA** – Pan-Atlantic; **NC** – Narrow continental; **NS** – The North Seitan; **TS** – Tethyan-Siberian; **ChCA** – Chinese-Central Asian endemics; **TNT** – Turkestanian-Northern Turanian endemics; **ITCA** – Irano-Turanian-Central Asian endemics; **KNTIT** – Kazakh-Northern Turanian, Irano-Turanian; **T** – Turanian endemics.

Entomologists (Kulumbetova 1998a, b, c, 1999; Khamraev 2003) discovered five new species: *Tarajalabrevicornis* (Reuter, 1879), *Emblethisdilaticollis* (Jakovlev, 1874), *Aethusnigronervosus* (Melichar, 1906), *Microporusvirgata* (Fabricius, 1974), and *Sciocorishelferi* (Fieber, 1851), which were indexed between 1998–2003 (Kulumbetova 1998a, b, c, 1999; Khamraev 2003) for the Lower Amudarya. These could be rare or migratory species, or are probably misidentified. These five species, shaded in Table 1, have not yet been verified and these records are not used in the distributional and zoogeographical analyses of the group; they are only mentioned in the checklist of the heteropterans found in the Khorezm region and Karakalpakstan.

Khamraev (2003) identified 110 species for the Khorezm, and Kulumbetova (1998a, b, c, 1999) 62 species for the Republic of Karakalpakstan.

The analysis of terrestrial Heteroptera in the Lower Amudarya by Gandjaeva (2007–2020) represented 39 species, which were first studied for the fauna of the Khorezm region and 87 species for the Republic of Karakalpakstan. According to the data, there are currently 149 species of terrestrial Heteroptera recorded in the Lower Amudarya (Fig. 2).

During 2007–2020, 149 species of terrestrial heteropterans were recorded in the Lower Amudarya territory as represented in Table 1.

From the surveys, it has been established that approximately 30 species are abundant and numerous. Sixty-two species are frequent, eight are sometimes encountered, and 49 were recorded as few. They belong to 17 families, 89 genera and the most numerous are Miridae – 37 species and Pentatomidae – 28 species, followed by Reduviidae – 15; Rhopalidae – 10; Geocoridae – 9; Rhyparochromidae – 8; Nabidae – 7; Coreidae, Lygaeidae, Cydnidae – 6, Anthocoridae – 4. Other families are represented by not more than two or three species (Table 2).

**Table 2.** Distribution of the number of genera, species within families, as well as their percentage (%) in the fauna of terrestrial heteropterans.

Family	Number of genera	%	Number of species	%
Anthocoridae	2	2.27	4	2.68
Nabidae	2	2.27	7	4.70
Miridae	20	21.59	37	24.16
Tingidae	3	3.41	3	2.01
Reduviidae	7	7.95	15	10.07
Alydidae	2	2.27	2	1.34
Coreidae	6	6.82	6	4.03
Rhopalidae	7	7.95	10	6.71
Stenocephalidae	1	1.14	2	1.34
Artheneidae	1	1.14	1	0.67
Geocoridae	3	3.41	9	6.04
Lygaeidae	5	5.68	6	4.70
Rhyparochromidae	5	5.68	8	5.37
Cydnidae	6	6.82	6	4.03
Pentatomidae	15	17.05	28	18.79
Scutelleridae	2	2.27	3	2.01
Pyrrhocoridae	2	2.27	2	1.34
**Total**:	**89**	**100**	**149**	**100**

**Figure 3.**
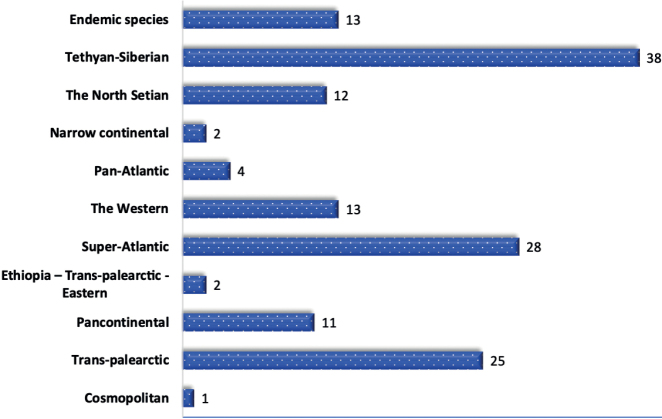
Species numbers of true bugs by area grouping.

Recorded species belong to 11 types according to sector ranges, and 28 groups of areas according to belt ranges (Table 3). The Lower Amudarya’s hemipteran species were divided into four large groups: Wide Areas, Holarctic Areas, Palaearctic Areas, and Endemic Areas (Fig. 3).

**Table 3.** Percentage of the terrestrial Heteroptera by area grouping.

Type area	The sector and belt range	Number of species	Species	Percentage
**I. Groups of wide areas**	**I.1. Cosmopolitan**	**1**	*Liorhyssushyalinus*;	**0.67**
**II. Holarctic**	**II.1. Trans-Palaearctic**	**9**		**6.04**
	a) Extratropical, Nearctic	3	*Lygusrugulipennis*, *Oriusalbidipennis*, *Derulalongipennis*;	2.01
	b) Boreal-subtropical, Nearctic	3	*Agnocorisrubicundus*, *Lyguspunctatus*, *Polymeruscognatus*;	2.01
	c) Boreal-subtropical	2	*Polymerusvulneratus*, *Orthotylusflavosparsus*;	1.34
	d) Boreal – subboreal	1	*Stenodematripsinosa*;	0.67
	**II.2. Pancontinental**	**1**		**0.67**
	a) Extratropical	1	*Deraeocorispunctulatus*;	0.67
**III. Palaearctic**	**III.1. Ethiopia – Trans-Palaearctic – Eastern**	**2**		**1.34**
	a) Southern	2	*Ectomocorisululans*, *Microporusnigrita*;	1.34
	**III.2. Trans-Palaearctic**	**16**		**10.74**
	a) Extratropical	1	*Europiellaalpina*;	0.67
	b) Arctic	3	*Brachynemagermari*, *Byrsinusfossor*, *Tarisafraudatrix*;	2.01
	c) Boreal	2	*Nabisferus*, *Nabissareptanus*;	1.34
	d) Boreal-subtropical	9	*Oriusniger*, *Adelphocorislineolatus*, *Lygusgemellatusgemellatus*, *Stenodemacalcaratum*, *Geocorisater*, *Coreusmarginatus*, *Brachycarenustigrinus*, *Corizushyoscyamihyoscyami*, *Rhopalusparumpunctatus*;	6.04
	e) Boreal-subboreal	1	*Lygaeusequestris*;	0.67
	**III.3. Super-Atlantic**	**28**		**18.79**
	a) Arcto-Subboreal	7	*Tuponiaelegans*, *Tuponiapallida*, *Coranusaegyptius*, *Nysiusgraminicolagraminicola*, *Emblethisgriseus*, *Emblethisverbasci*, *Corizuslimbatus*;	4.70
	b) Boreal-subboreal	1	*Orthopsbasalis*;	0.67
	c) Boreal-subtropical	7	*Lyguspratensis*, *Notostiraelongata*, *Eurydemaornata*, *Eurydemaoleracae*, *Palomenaprasina*, *Orthopskalmi*, *Chorosomaschillingi*;	4.70
	d) Subboreal	8	*Nabisrugosus*, *Nabisviridis Brullé*, *Heterocapillustigripes*, *Macrotylusherrichi*, *Monosteiradiscoidalis*, *Beosusquadripunctatus*, *Codophilavaria*, *Camptopuslateralis*;	5.37
	e) Subboreal-subtropical	2	*Eurydemaventralis*, *Graphosomalineatum*;	1.34
	f) Southern	3	*Anthocorispilosus*, *Oxycarenuspallens*, *Emblethisciliatus*;	2.01
	**III. 4. The Western**	**13**		**8.72**
	a) Boreal	2	*Deraeocorisserenus*, *Adelphocorisseticornis*;	1.34
	b) Boreal-subtropical	5	*Lamprodemamaura*, *Stictopleurusunicolor*, *Sehirusmorio*, *Aeliaacuminata*, *Carpocorisfuscispinus*;	3.36
	c) Boreal-subboreal	2	*Pyrrhocorisapterus*, *Megaloceroearecticornis*;	1.34
	d) Subboreal	3	*Artheneisalutacea*, *Brachycoleusdecolor*, *Geocorisdispar*;	2.01
	e) Southern	1	*Henestarishalophilus*;	0.67
	**III. 5. Pan-Atlantic**	**4**		**2.68**
	a) Boreal-subtropical	2	*Stenodemalaevigata*, *Campylommaverbasci*;	1.34
	b) Boreal-subboreal	1	*Trigonotylusruficornis*;	0.67
	c) Subboreal-subtropical	1	*Prostemmasanguineum*;	0.67
	**III. 6. Pancontinental**	**10**		**6.71**
	a) Northern	1	*Geocorislapponicus*;	0.67
	b) Boreal-subtropical	5	*Ortholomuspunctipennis*, *Emblethisdenticollis*, *Holcostethusstrictusvernalis*, *Carpocorispudicus*, *Trigonotyluspulchellus*;	3.36
	c) Subboreal	1	*Oriusribauti*;	0.67
	d) Subboreal-subtropical	1	*Eurygasterintegriceps*;	0.67
	e) Southern	2	*Campylommaannulicorne*, *Stephanitispyri*;	1.34
	**III. 7. Narrow Continental**	**2**		**1.34**
	a) Eastern Mediterranean Gobian	1	*Stenodematuranica*;	0.67
	b) Mediterranean-Irano-Turanian	1	*Geocorisfedtschenkoi*;	0.67
	**III. 8. The North Setian**	**12**		**8.05**
	a) Trans-Scythian	1	*Geocorisarenarius*;	0.67
	b) Western Scythian	3	*Coranussubapterus*, *Campylommadiversicorne*, *Camptotylusmeyeri*;	2.01
	c) Eastern Scythian	8	*Corizustetraspilus*, *Megalotomusornaticeps*, *Desertomenidaquadrimaculata*, *Cellobiusabdominalis*, *Eurydemawilkinsi*, *Eurydemamaracandica*, *Tarisavirescens*, *Scantiusaegyptius*;	5.37
	**III. 9. Tethyan-Siberian**	**38**		**25.50**
	a) Western-Scythian-Saharo-Gobian	1	*Stenolemusbogdanovi*;	0.67
	b) Euro-Mediterranean – Turanian	10	*Spilostethuspandurus*, *Tarisapallescens*, *Reduviustestaceus*, *Centrocorisvolxemi*, *Bathysolennubilus*, *Coriomerisvitticollis*, *Rhopalusdistinctus*, *Engistusexsanguis*, *Aeliafurcula*, *Graphosomaconsimile*;	6.71
	c) Irano-Turanian-Gobian	4	*Megacoelumbrevirostre*, *Orthotyluseleagni*, *Oncocephalusbrachymerus*, *Bothrostethusannulipes*;	2.68
	d) Irano-Turanian	15	*Reduviusdisciger*, *Reduviuschristophi*, *Engistussalinus*, *Tarisaelevata*, *Desertomenidaalbula*, *Odontotarsusimpictus*, *Odontotarsusangustatus*, *Amaurocoriscandidus*, *Aeliamelanota*, *Dolycorispenicillatus*, *Apodiphusintegriceps*, *Menaccarusdeserticola*, *Maccevethuscorsicuspersicus*, *Dicranomerusmarginatus*, *Dicranomerusferghanensis*;	10.07
	e) Kazakh-Northern Turanian, Irano-Turanian	6	*Nabispalifer*, *Rhynocorismonticolamonticola*, *Rhynocorisnigronitens*, *Spilostethusrubriceps*, *Bleteogonusbeckeri*, *Geocorisscutellatus*;	4.03
	f) Tethys-Ethiopian	2	*Hyalocorispilicornis*, *Aethuspilosulus*;	1.34
**IV. Endemics**	**IV. Endemics**	**13**		**8.72**
	a) Chinese-Central Asian	2	*Nabisremanei*, *Tuponiaroseipennis*;	1.34
	b) Chinese-Irano-Central Asian	1	*Reduviusfedtschenkianus*;	0.67
	c) Turkestanian-Northern Turanian	6	*Stibaropushohlbecki*, *Holcostethusnitidus*, *Lyguspachycnemis*, *Camptotylideaalba*, *Reduviussemenovi*, *Reduviuselegans*;	4.03
	d) Irano-Turanian-Central Asian	3	*Vachiriadeserta*, *Tingisleptochila*, *Oncocephalustermezanus*;	2.01
	e) Turanian	1	*Enoplopseversmanni*;	0.67
**Total**:		**149**		**100**

- Broad areas – extend beyond the Holarctic;
- Cosmopolitan areas – occur on at least three continents;
- Holarctic areas – cover the Palaearctic and the Nearctic region;
- Palaearctic areas – cover parts of Europe, Asia, and North Africa;
- Nearctic areas – cover North America, Mexico, and Greenland;
- The Ethiopia – Trans-Palaearctic – Eastern areas – this complex combines the Palaearctic, Ethiopia and Eastern regions;
- Trans-Palaearctic areas – cover the entire Palaearctic;
- Super-Atlantic areas – cover from the Atlantic sectors to the Eastern transitional sectors;
- The Western areas – cover the part of the Palaearctic Realm from the Eastern Atlantic to the Western Eucontinental sectors;
- Pan-Atlantic areas – encompass the Atlantic sector as well as the western subcontinental subsectors;
- Pancontinental areas – located from the sub-Atlantic to the eastern continental sectors inclusive;
- Narrow Continental areas – cover the Sahara-Gobi Desert area, the Mediterranean and the Irano-Turanian sub-areas.
- The North-Setian areas – cover the Trans-Scythian, the Western-Scythian, and the Eastern-Scythian sub-regions;
- Tethyan-Siberial areas – cover the Tethyan Subkingdom, Scythian, Setian, and European, Mediterranean, and Irano-Turanian subregions;
- Endemic areas – occur only in a certain area and nowhere else.

In the northern part of Uzbekistan, only one species (0.67%) is cosmopolitan. The group of the Holarctic range is characterized by no more than ten species, which constitutes 6.71% of the total, and most species belong to the Palaearctic group, which is most diverse. The group contains 125 species (83.89%), with 38 from the Tethyan-Siberian type constituting 25.50%. Approximately 15 species account for 10.07% of the Irano-Turanian range, while ten species constitute 6.71% of the Euro-Mediterranean-Turanian range. In the Super-Atlantic range, 28 species account for 18.79%, with eight species making up 5.37% of subboreal and seven species accounting for 4.70% of boreal-subtropical species recorded. Sixteen Trans-Palaearctic species (10.74%) have been recorded, followed by 13 Western (8.72%), 12 North Setian (8.05%), ten Pancontinental (6.71%), and four Pan-Atlantic (2.68%) species. The number of species with Ethiopia-Trans-Palaearctic-Eastern distributions and Narrow Continental is only two for each area or 1.34%. It can be seen that the prevailing part of the group,125 species (83.89%), were found in wider areas of the Holarctic, and 13 are endemic species (8.72%).

The endemics are divided into Chinese-Central Asian, Chinese-Irano-Central Asian, Turkestanian-Northern Turanian, Irano-Turanian-Central Asian, and Turanian (found in Central Asia only). For the assessment of any territory, endemics have a high conservation value since they indicate the distinctive nature of the fauna.

## ﻿Conclusions

In this study, we collected new 39 species for the Khorezm region and 87 species for the Republic of Karakalpakstan during 2007–2020. In addition, we compare our collections with reports of Khamraev (2003) and Kulumbetova (1998a, b, c, 1999) and a total of 154 species (17 families) of terrestrial Heteroptera (Fig. 2, Table 1) were analyzed.

Khamraev (2003) identified 110 species for the Khorezm, and Kulumbetova (1998a, b, c, 1999) 62 species for the Republic of Karakalpakstan. There are currently 149 species of terrestrial Heteroptera in the Lower Amudarya. The results show that 62 species are highly abundant at the site, divided into 17 families and 89 genera, with the Miridae and Pentatomidae having most species (37 and 28, respectively), followed by Reduviidae (15), Rhopalidae (10), Geocoridae (9), Rhyparochromidae (8), Nabidae (7), Coreidae, Lygaeidae, Cydnidae (6 each) (Table 2).

The Heteroptera fauna of Khorezm and Karakalpakstan can be divided into four groups: Cosmopolitan with one species (0.67%); Holarctic, with no more than ten species, or 6.71%; Palaearctic, with most of species (125 species, or 83.89%); and endemic with 13 species, or 8.72%.

An understanding of the fauna is important, as the productivity of crops is currently being negatively impacted by invasive species from neighboring countries. For example, recently we recorded (Gandjaeva et al. 2022b) the brown marmorated stink bug *Halyomorphahalys* (Stål, 1855) (Heteroptera: Pentatomidae) from Uzbekistan for the first time. Several adults and immatures were found in the Khorezm and Ferghana provinces. This species is native to East Asia (China, Korea, Japan, and Taiwan) (Rider et al. 2002; Hoebeke and Carter 2003; Rider 2006, 2016) and is a dangerous pest of many agricultural plants. Therefore, more study is required to examine the impacts of dispersion in a northern environment. In the north-western region of Uzbekistan, an increase in the number of invasive Heteroptera is expected, which will require careful monitoring.
